# Distinct effects of EGFR inhibitors on epithelial- and mesenchymal-like esophageal squamous cell carcinoma cells

**DOI:** 10.1186/s13046-017-0572-7

**Published:** 2017-08-01

**Authors:** Masahiro Yoshioka, Shinya Ohashi, Tomomi Ida, Yukie Nakai, Osamu Kikuchi, Yusuke Amanuma, Junichi Matsubara, Atsushi Yamada, Shin’ichi Miyamoto, Mitsuteru Natsuizaka, Hiroshi Nakagawa, Tsutomu Chiba, Hiroshi Seno, Manabu Muto

**Affiliations:** 1 Department of Gastroenterology and Hepatology, Kyoto University Graduate School of Medicine, 54 Kawaharacho, Shogoin, Sakyo-ku, Kyoto, 606-8507 Japan; 2 Department of Therapeutic Oncology, Kyoto University Graduate School of Medicine, 54 Kawaharacho, Shogoin, Sakyo-ku, Kyoto, 606-8507 Japan; 3 Department of Medical Oncology, Dana-Farber Cancer Institute, 450 Brookline Avenue, Boston, MA 02215 USA; 4 Department of Gastroenterology and Hepatology, Hokkaido University Hospital, N14W5, Kita-ku, Sapporo, 060-8468 Japan; 5 Gastroenterology Division, Department of Medicine, University of Pennsylvania, 3101 Walnut St, Philadelphia, PA 19104 USA; 6 Kansai Electric Power Hospital, 2-1-7 Fukushima, Fukushima-ku, Osaka, 553-0003 Japan

**Keywords:** Esophageal squamous cell carcinoma, EGFR inhibitor, Cell differentiation, Epithelial-like cell, Mesenchymal-like cell

## Abstract

**Background:**

Epidermal growth factor receptor (EGFR) plays a pivotal role in the pathophysiology of esophageal squamous cell carcinoma (ESCC). However, the clinical effects of EGFR inhibitors on ESCC are controversial. This study sought to identify the factors determining the therapeutic efficacy of EGFR inhibitors in ESCC cells.

**Methods:**

Immortalized-human esophageal epithelial cells (EPC2-hTERT), transformed-human esophageal epithelial cells (T-Epi and T-Mes), and ESCC cells (TE-1, TE-5, TE-8, TE-11, TE-11R, and HCE4) were treated with the EGFR inhibitors erlotinib or cetuximab. Inhibitory effects on cell growth were assessed by cell counting or cell-cycle analysis. The expression levels of genes and proteins such as involucrin and cytokeratin13 (a squamous differentiation marker), E-cadherin, and vimentin were evaluated by real-time polymerase chain reaction or western blotting. To examine whether mesenchymal phenotype influenced the effects of EGFR inhibitors, we treated T-Epi cells with TGF-β1 to establish a mesenchymal phenotype (mesenchymal T-Epi cells). We then compared the effects of EGFR inhibitors on parental T-Epi cells and mesenchymal T-Epi cells. TE-8 (mesenchymal-like ESCC cells)- or TE-11R (epithelial-like ESCC cells)-derived xenograft tumors in mice were treated with cetuximab, and the antitumor effects of EGFR inhibitors were evaluated.

**Results:**

Cells were classified as epithelial-like or mesenchymal-like phenotypes, determined by the expression levels of E-cadherin and vimentin. Both erlotinib and cetuximab reduced cell growth and the ratio of cells in cell-cycle S phase in epithelial-like but not mesenchymal-like cells. Additionally, EGFR inhibitors induced squamous cell differentiation (defined as increased expression of involucrin and cytokeratin13) in epithelial-like but not mesenchymal-like cells. We found that EGFR inhibitors did not suppress the phosphorylation of EGFR in mesenchymal-like cells, while EGFR dephosphorylation was observed after treatment with EGFR inhibitors in epithelial-like cells. Furthermore, mesenchymal T-Epi cells showed resistance to EGFR inhibitors by circumventing the dephosphorylation of EGFR signaling. Cetuximab consistently showed antitumor effects, and increased involucrin expression in TE-11R (epithelial-like)-derived xenograft tumors but not TE-8 (mesenchymal-like)-derived xenograft tumors.

**Conclusions:**

The factor determining the therapeutic effects of EGFR inhibitors in ESCC cells is the phenotype representing the epithelial-like or mesenchymal-like cells. Mesenchymal-like ESCC cells are resistant to EGFR inhibitors because EGFR signaling is not blocked. EGFR inhibitors show antitumor effects on epithelial-like ESCC cells accompanied by promotion of squamous cell differentiation.

**Electronic supplementary material:**

The online version of this article (doi:10.1186/s13046-017-0572-7) contains supplementary material, which is available to authorized users.

## Background

Esophageal squamous cell carcinoma (ESCC) is one of the most aggressive cancers with a poor prognosis despite recent advances in therapeutics [1]. Among the biochemical cascades that regulate squamous keratinocyte biology, epidermal growth factor receptor (EGFR) signaling is one of the key molecular pathways involved in the regulation of cell proliferation, cell differentiation, and tumorigenesis [2–4]. In addition, EGFR overexpression is found in 59.6%–76% of patients with ESCC [5] and is associated with a poor prognosis [6]. Thus, EGFR signaling plays a pivotal role in the pathophysiology of ESCC.

Tyrosine kinase inhibitors and monoclonal antibodies that inhibit EGFR signaling have been developed as EGFR inhibitors [7] and have proven effective in the treatment of various cancers including non-small cell lung cancer, colorectal cancer, pancreatic cancer, and squamous cell cancer of the head and neck [8–11]. Although several clinical studies show the beneficial effects of EGFR inhibitors in the treatment of ESCC [12, 13], evidence supporting the validity of EGFR-targeting therapies for esophageal cancer [14] is not robust. Therefore, we suspect that some factors influence the therapeutic efficacy of EGFR inhibitors in ESCC.

Recently, EGFR signaling blockade has been shown to promote squamous cell differentiation in skin keratinocytes as well as cutaneous SCCs [2]. The concept of differentiation therapy for SCC was first proposed by Pierce et al. in 1971 [15]. They noted that the growth of SCC is dependent upon the proliferation of undifferentiated but not differentiated cancer cells, because well-differentiated cancer cells cannot synthesize DNA or form tumors on transplantation [15]. The inhibition of squamous cell differentiation in transformed-esophageal epithelial cells consistently accelerates tumor development [16]. Moreover, the promotion of squamous cell differentiation in skin, lung, and head and neck SCCs suppresses the growth of tumor cells [17]. Thus, a strategy targeting “squamous cell differentiation” could be a potential new therapy for SCCs, although its effects on ESCCs remain unknown. Here, we postulate that the promotion of squamous cell differentiation by EGFR inhibitors may suppress the growth of ESCC cells, which would contribute to establishing “differentiation therapy” for ESCC.

In this study, we treated various human esophageal epithelial cells including immortalized- or transformed-esophageal epithelial cells and human ESCC cells with EGFR inhibitors and identified factors that could explain the inhibitory effects of EGFR signaling on these cells.

## Methods

### Cells

Immortalized-human esophageal epithelial cells (EPC2-hTERT) [18] and derivatives transformed by either EGFR and p53^R175H^ (T-Epi) [19] or SV40 large T antigen and Ha-RasV12 (T-Mes) [20], fetal esophageal fibroblast cells (FEF3) [21], and ESCC cells (HCE4, TE-1, TE-5, TE-8, and TE-11) were described previously [18–21]. EPC2-hTERT, T-Epi, T-Mes, HCE4, and FEF3 cells were provided by the Cell Culture Core of the University of Pennsylvania [18–21]. ESCC cells (TE-1, 5, 8, and 11) were obtained from Riken BioResource Center (Ibaraki, Japan) [22]. These ESCC cells reportedly do not harbor an EGFR mutation [23]. TE-11R cells were 5-FU-resistant cells established via exposure to TE-11 cells with incremental concentrations of 5-FU [24, 25]. EPC2-hTERT cells and their derivatives were grown in keratinocyte serum-free medium (KSFM; Thermo Fischer Scientific, Waltham, MA) supplemented with 100 μg/mL of streptomycin and 100 units/mL of penicillin (Thermo Fischer Scientific) at 37 °C in a 5% CO_2_ incubator, as previously described [18–20]. TE-series or HCE-4 cells were cultured in RPMI1640 medium or DMEM (both Thermo Fischer Scientific), respectively, supplemented with 10% fetal bovine serum (Thermo Fischer Scientific), 100 μg/mL of streptomycin, and 100 units/mL of penicillin (Thermo Fischer Scientific) at 37 °C in a 5% CO_2_ incubator. A Countess Automated Cell Counter (Thermo Fischer Scientific) was used to count cells with 0.2% trypan blue dye to exclude dead cells. Phase-contrast images were acquired using a Nikon Eclipse Ti-S microscope.

### Cell treatment

Cells were treated with 1 μM erlotinib (a tyrosine kinase inhibitor; Cell Signaling Technology, Danvers, MA) reconstituted in 100 μg/mL DMSO or cetuximab (a monoclonal antibody; Merck, Darmstadt, Germany), 20 ng/mL recombinant EGF (rEGF; Thermo Fischer Scientific), recombinant human TGF-β1 (R&D Systems Inc., Minneapolis, MN) reconstituted in 4 mmol/L HCl containing 0.1% bovine serum albumin or DAPT (Sigma-Aldrich) reconstituted in DMSO.

Cells were counted to analyze cell growth with or without EGFR inhibitors. Cells 2 × 10^5^ were disseminated into 12-well plates (EPC2-hTERT, T-Epi, T-Mes) or 5 × 10^5^ into 100-mm dishes (TE-1, TE-5, TE-8, TE-11, TE-11R, HCE-4, and mesenchymal T-Epi). Erlotinib or cetuximab was added to the medium, and cell numbers were counted every 24 h. Each experiment was performed in triplicate.

To examine the effects of EGFR activation on esophageal cells, 20 ng/mL rEGF was added to the medium. EPC2-hTERT, T-Epi, and T-Mes cells (1 × 10^6^ cells) were plated in 60-mm dishes and cultured overnight before being treated with rEGF for 48 h.

To induce epithelial-to-mesenchymal transition in T-Epi cells, 5 ng/mL recombinant human TGF-β1 was added to the medium [26, 27]. T-Epi cells (1 × 10^6^) were plated in 100-mm dishes and cultured with TGF-β1. Medium containing TGF-β1 was changed every 2 days. Cells were passaged once they reached 70% confluence. T-Epi cells were treated with recombinant TGF-β1 for 14 days.

### RNA isolation, cDNA synthesis, and real-time reverse transcriptase-PCR

RNA extraction and cDNA synthesis were conducted as previously described [16, 26]. Real-time reverse transcriptase polymerase chain reaction (RT-PCR) was performed with the LightCycler 480 Instrument II Real-Time PCR System (Roche Diagnostics Ltd., Rotkreuz, Switzerland). The relative expression of each mRNA was normalized to β-actin as an internal control. The primers used in this study were as follows: *IVL*: forward 5′-TCCTCCAGTCAATACCCATCAG-3′; reverse 5′-CAGCAGTCATGTGCTTTTCCT-3′; *CK13*: forward 5′-CCCCAGGCATTGACCTGAC-3′; reverse 5′-GTGTTGGTAGACACCTCCTTG-3′; *ACTB*: forward 5′-TTGTTACAGGAAGTCCCTTGCC-3′; reverse: 5′-ATGCTATCACCTCCCCTGTGTG-3′.

### Western blotting

Whole-cell lysates were prepared as described previously [26, 27]. Briefly, cells were washed twice with ice-cold phosphate-buffered saline and lysed with a RIPA (Nacalai Tesque, Kyoto, Japan). After 30 min on ice, the cell lysates were centrifuged at 14000 rpm at 4 °C for 30 min. Protein concentrations were determined by BCA protein assay (Pierce Biotechnology, Rockford, IL). Protein (10–15 μg) was heat-denatured in Sample Buffer Solution with Reducing Reagent (6×) for SDS-PAGE (Nacalai Tesque) at 70 °C for 10 min. The protein samples were separated on Mini-PROTEAN® TGX™ Gels (BIO-RAD Laboratories Inc., Hercules, CA) and transferred to a polyvinylidene difluoride membrane (Trans-Blot® Turbo™ Transfer Pack, BIO-RAD). The membrane was blocked in 5% nonfat milk and 1% bovine serum albumin (BSA, pH 5.2, Fraction V, Wako Pure Chemical Industries, Osaka, Japan) in TBS-T (10 mM Tris, 150 mM NaCl, pH 8.0, and 0.1% Tween 20) for 1 h at room temperature. Membranes were probed with the primary antibody diluted in 5% nonfat milk and 1% BSA in TBS-T overnight at 4 °C, washed 3 times in TBS-T, incubated with secondary antibody in 5% nonfat milk and 1% BSA in TBS-T for 1 h at room temperature, and finally washed 3 times in TBS-T. The signal was visualized by an enhanced chemiluminescence solution (SuperSignal® West Femto Maximum Sensitivity Substrate, Thermo Scientific or Immobilon™ Western Chemiluminescent HRP Substrate, Merck Millipore) and exposed to ChemiDoc™ Touch Imaging System (BIO-RAD). Densitometric analyses of Western blot bands were performed using Image Lab™ Software (BIO-RAD). Data was calibrated with β-actin as a loading control in arbitrary units.

Primary antibodies and the titers used in this study were as follows: rabbit monoclonal anti-EGFR antibody (D38B1, #4267, Cell Signaling Technology, Inc., Danvers, MA; 1:1000), rabbit monoclonal anti-phospho-EGFR antibody (Tyr1068) (D7A5, #3777, Cell Signaling; 1:1000), rabbit monoclonal anti-vimentin antibody (D21H3, #5741, Cell Signaling; 1:1000), rabbit monoclonal anti-E-cadherin antibody (24E10, #3195, Cell Signaling; 1:1000), mouse monoclonal anti-involucrin antibody (SY5, I9018, Sigma-Aldrich; 1:3000), and rabbit monoclonal anti-β-actin antibody (13E5, #5125, Cell Signaling; 1:5000). The secondary antibodies and titers were anti-rabbit IgG, HRP-linked whole Ab donkey (NA-934, GE Healthcare, 1:2000) and anti-mouse IgG, HRP-linked whole Ab sheep (NA-931, GE Healthcare, 1:2000).

### Flow cytometry

Click-iT EdU Flow Cytometry Assay Kit (Invitrogen) was used to assess the effects of EGFR inhibitors on the cell cycle. After cells were cultured in the presence or absence of EGFR inhibitors for 72 h, EGFR inhibitors were removed and the cells were incubated with Click-iT EdU, harvested, and treated according to the manufacturer’s instructions. Cells were analyzed by flow cytometry (BD LSRFortessa™ cell analyzer; BD Biosciences) and the data was analyzed using BD FACSDiva software (BD Biosciences). The percentages of cells in S-phase were determined; cells in a proliferating population (S phase) show high fluorescence intensity (p3), whereas cells in non-proliferating populations show low fluorescence intensity (p2).

### *In vivo* experiments

All experiments conformed to the relevant regulatory standards and were approved by the Institutional Animal Care and Use Committee of Kyoto University (Med Kyo 15330).

Xenograft transplantation was performed as described previously [24]. Here, we used two ESCC cells (TE-8 and TE-11R) because other ESCC cells (TE-1, TE-5, TE-11, and HCE4) did not form xenograft tumors on athymic nude mice. Briefly, TE-11R cells (1 × 10^7^) and TE-8 cells (4 × 10^6^) were suspended in 50% Matrigel (BD Biosciences, San Jose, CA), followed by subcutaneous implantation into the left flank of 9-week-old nude male mice (CLEA Japan, Inc., Tokyo, Japan). Xenografted tumors were used for the following experiments and divided into two groups when they reached a volume of about 300–1000 mm^3^ at 70 days (TE-11R) or 25 days (TE-8) after injection. Cetuximab (50 mg/kg) or PBS was administered intraperitoneally. The first day of administration was defined as day 0, and cetuximab was administered on days 0, 4, and 7. The tumors were monitored twice a week with a caliper, and tumor volume (mm^3^) was calculated using the following formula: (length) × (width)^2^ × 0.5. On day 11, mice were painlessly sacrificed by inhalation of isoflurane (Escain, Mylan Pharmaceuticals, Tokyo, Japan) and cervical dislocation. Tissue samples were fixed in 10% neutral buffered formalin (Wako Pure Chemical Industries, Ltd.) overnight, embedded in paraffin, and cut into 4 µm sections for standard hematoxylin and eosin (H&E) staining and immunohistochemistry.

### Immunohistochemistry

Tyramide signal amplification avidin–biotin complex method was used for immunohistochemistry [28]. Incubation and washing procedures were carried out at room temperature unless otherwise stated. After deparaffinization and antigen retrieval by incubation in 0.1% Trypsin solution at 37 °C for 30 min, endogenous peroxidase activity was blocked by 0.3% H_2_O_2_ in methyl alcohol for 30 min. The glass slides were washed in PBS (6 times, 5 min each) and mounted with 1% horse normal serum in PBS for 30 min. The primary antibody, mouse monoclonal anti-involucrin antibody (SY5, I9018, Sigma-Aldrich; 1:150), was subsequently applied overnight at 4 °C. Cells were incubated with biotinylated horse anti-mouse serum (second antibody, VECTOR lab) diluted to 1:300 in PBS for 40 min, and followed by PBS washes (6 times, 5 min). Avidin-biotin-peroxidase complex (ABC) (ABC-Elite, Vector Laboratories, Burlingame, CA) diluted 1:100 in BSA was applied for 50 min. After washing in PBS (6 times, 5 min), a coloring reaction was carried out with DAB, and nuclei were counterstained with hematoxylin.

### Statistical analyses

Data are presented as the means ± standard deviation of triplicate experiments, unless otherwise stated. The 2-tailed Student’s t-test between two groups was selected for data analysis. *P* < 0.05 was considered statistically significant. All statistical analyses were performed using SPSS 21 for Windows (SPSS Inc., Chicago, IL).

## Results

### Effects of EGFR inhibitors on cell growth and squamous cell differentiation in immortalized-human esophageal epithelial cells

**Fig. 1:**
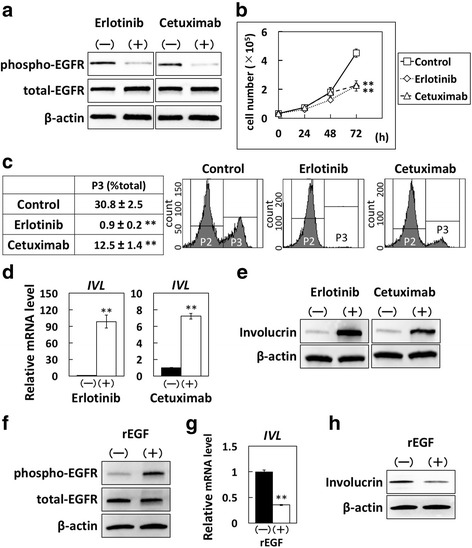
Effects of EGFR inhibitors on immortalized-human esophageal epithelial cells. EPC2-hTERT, an immortalized-human esophageal epithelial cell line, was treated with erlotinib (1 μM) or cetuximab (100 μg/mL). **a** Phosphorylated- and total-EGFR protein levels in EPC2-hTERT cells treated with or without erlotinib or cetuximab for 24 h, determined by western blotting. **b** Cell growth of EPC2-hTERT cells treated with or without erlotinib or cetuximab for 72 h, determined by cell count. Results are presented as means ± SD (bars). (** *p* < 0.01 vs. vehicle control; *n* = 3). **c** Cell cycle analysis of EPC2-hTERT cells treated with or without erlotinib or cetuximab, analyzed by EdU assay. Cells in S phase are plotted in p3, and cells in other phases in p2. The experiments were conducted in triplicate, and results are presented as means ± SD. Representative data are shown. (** *p* < 0.01 vs. vehicle control; *n* = 3). **d** Involucrin mRNA expression levels in EPC2-hTERT cells treated with or without erlotinib or cetuximab for 72 h. The mRNA levels for the *IVL* gene relative to the untreated cells were determined by QPCR. The gene for β-actin served as an internal control. (**p < 0.01 vs. vehicle control; *n* = 3). **e** Involucrin protein production levels in EPC2-hTERT cells treated with or without erlotinib or cetuximab for 72 h, determined by western blotting. **f** Phosphorylated- and total-EGFR protein levels in EPC2-hTERT cells treated with human recombinant EGF (rEGF) (20 ng/mL) for 48 h, determined by western blotting. **g** Involucrin mRNA expression levels in EPC2-hTERT cells treated with rEGF for 48 h, determined by QPCR. (**p < 0.01 vs. vehicle control; *n* = 3). **h** Involucrin protein production levels in EPC2-hTERT cells treated with rEGF for 48 h, determined by western blotting

To examine the effects of EGFR inhibitors on cell growth and/or squamous cell differentiation in immortalized-human esophageal epithelial cells, EPC2-hTERT cells were treated with the EGFR inhibitors erlotinib or cetuximab. The phosphorylation of EGFR was suppressed (Fig. 1a) by treatment with erlotinib (1 μM) or cetuximab (100 μg/mL). We then assessed the inhibitory effects of EGFR inhibitors on EPC2-hTERT cell growth. Treatment with erlotinib or cetuximab significantly suppressed cell growth in these cells (Fig. 1b), and EdU assay showed that both erlotinib and cetuximab reduced the ratio of cells in S phase, the DNA replication phase of the cell cycle (Fig. 1c). Next, we examined the effects of EGFR inhibition on squamous cell differentiation by assessing the expression levels of squamous cell differentiation markers, involucrin and CK13. After treatment for 72 h, both erlotinib and cetuximab increased the expression of involucrin mRNA (Fig. 1d) and protein (Fig. 1e) as well as the expression of CK13 mRNA (Additional file 1 Figure S1A). Thereafter, we treated EPC2-hTERT cells with 20 ng/mL rEGF to investigate the effects of EGFR signal activation in EPC2-hTERT cells. The phosphorylation of EGFR was enhanced by treatment with rEGF (Fig. 1f). The expression levels of involucrin mRNA and protein (Figs. 1g, h) and those of CK13 mRNA (Additional file 1 Figure S1B) were reduced by treatment with rEGF.

### Distinct effects of EGFR inhibitors on epithelial- and mesenchymal-like transformed-human esophageal epithelial cells

**Fig. 2:**
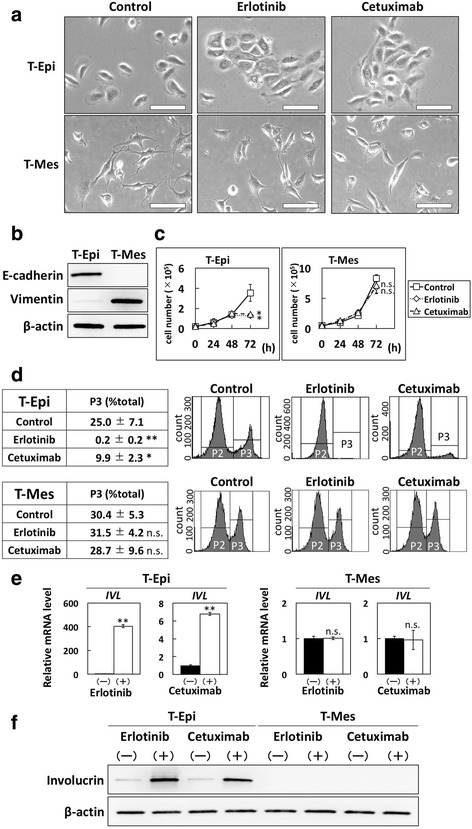
Effects of EGFR inhibitors on cell growth and squamous cell differentiation in transformed-human esophageal epithelial cells. **a** Phase-contrast images of T-Epi and T-Mes cells treated with vehicle control, erlotinib, or cetuximab for 72 h. Treatment with erlotinib or cetuximab induced cell-cell contact in T-Epi cells but not T-Mes cells. Scale bar, 40 μm. **b** E-cadherin and vimentin protein levels in transformed-human esophageal epithelial cells, T-Epi and T-Mes, determined by western blotting. **c** Cell growth of T-Epi or T-Mes cells treated with or without erlotinib or cetuximab for 72 h, determined by cell count. Results are presented as means ± SD (bars). (* *p* < 0.05 vs. vehicle control; n.s., not significant, *n* = 3). **d** Cell cycles of T-Epi and T-Mes cells treated with or without erlotinib or cetuximab for 72 h, analyzed by EdU assay. The experiments were conducted in triplicate, and results are presented as means ± SD. Representative data are shown. (*p < 0.05 vs. vehicle control; ** *p* < 0.01 vs. vehicle control; n.s., not significant; *n* = 3). **e** Involucrin mRNA expression levels in T-Epi or T-Mes cells treated with or without erlotinib or cetuximab for 72 h, determined by QPCR. (**p < 0.01 vs. vehicle control; n.s., not significant; *n* = 3). **f** Involucrin protein levels in T-Epi or T-Mes cells treated with or without erlotinib or cetuximab, determined by western blotting

Next, we examined the effects of EGFR inhibitors in transformed-human esophageal epithelial cells. Here, we used two cell lines, T-Epi and T-Mes, which are established transformed-human esophageal epithelial cells [19, 20]. As shown in Fig. 2a, T-Epi cells were round as seen in epithelial cells and T-Mes cells had a spindle-like morphology as seen in mesenchymal cells. To characterize these cells as either epithelial or mesenchymal phenotypes, we examined the expression levels of E-cadherin (epithelial marker) and vimentin (mesenchymal marker). Consistent with their morphology, T-Epi cells showed high expression of E-cadherin and low expression of vimentin, whereas T-Mes cells showed the reverse (Fig. 2b). Accordingly, T-Epi cells could be categorized as epithelial-like esophageal cells, and T-Mes cells as mesenchymal-like esophageal cells. When these cells were treated with erlotinib or cetuximab for 72 h, cell-cell contact was observed in T-Epi cells but not T-Mes cells (Fig. 2a). This result indicates that the effects of EGFR inhibition on epithelial- and mesenchymal-like esophageal cells might be different.

Erlotinib and cetuximab significantly suppressed the growth of T-Epi cells but not T-Mes cells (Fig. 2c). EdU assay showed that both erlotinib and cetuximab reduced the ratio of T-Epi cells in S phase; however, T-Mes cells were unaffected (Fig. 2d). Next, we investigated the expression levels of involucrin and CK13 in T-Epi and T-Mes cells treated with EGFR inhibitors. Similar to the events in EPC2-hTERT cells treated with EGFR inhibitors, both erlotinib and cetuximab increased the expression of involucrin mRNA (Fig. 2e) and protein (Fig. 2f), and CK13 mRNA (Additional file 2 Figure S2A) in T-Epi cells. In contrast, neither mRNA nor protein expression was altered in T-Mes cells (Figs. 2e, f, Additional file 2 Figure S2A).

Furthermore, we investigated the effects of EGFR activation on cells treated with rEGF and found that the phosphorylation of EGFR was increased in T-Epi cells but not T-Mes cells (Additional file 2 Figure S2B). The expression of involucrin mRNA and protein (Additional file 2 Figure S2C, D) and that of CK13 mRNA (Additional file 2 Figure S2E) were decreased by treatment with rEGF in T-Epi cells but not T-Mes cells (Additional file 2 Figure S2C, D, E). Moreover, inhibitory effects of squamous cell differentiation in rEGF-treated T-Epi cells were counteracted when erlotinib was added together with rEGF. Here, rEGF or erlotinib did not affect EGFR signaling or squamous cell differentiation in T-Mes cells (Additional file 2 Figure S2B, C, D, E).

### Distinct effects of EGFR inhibitors on epithelial- and mesenchymal-like ESCC cells

**Fig. 3:**
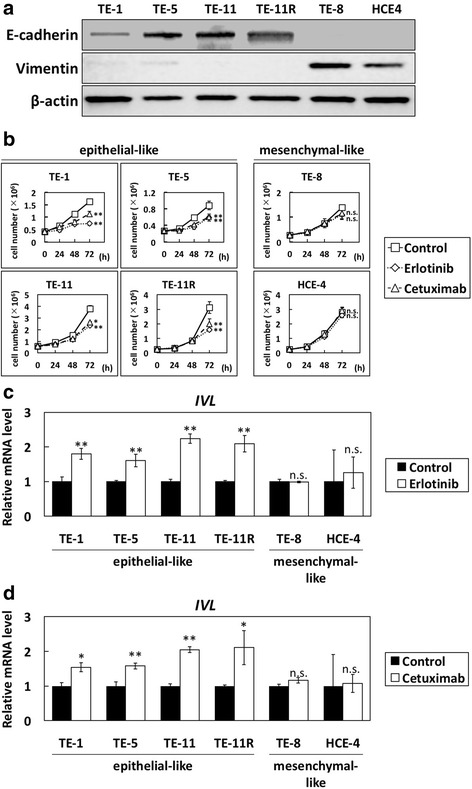
Effects of EGFR inhibitors on cell growth and squamous cell differentiation in ESCC cells. **a** E-cadherin and vimentin protein levels in ESCC cells were determined by western blotting. TE-1, TE-5, TE-11, and TE-11R cells were considered epithelial-like ESCC cells, while TE-8 and HCE4 were classed as mesenchymal-like ESCC cells. **b** Cell growth of epithelial- and mesenchymal-like ESCC cells treated with or without erlotinib or cetuximab for 72 h, determined by cell count. Results are presented as means ± SD (bars). (*p < 0.05 vs. vehicle control; ** *p* < 0.01 vs. vehicle control; n.s., not significant; *n* = 3). **c** Involucrin mRNA levels in ESCC cells treated with erlotinib for 72 h, determined by QPCR. (**p < 0.01 vs. vehicle control; n.s., not significant; *n* = 3). **d** Involucrin mRNA levels in ESCC cells treated with cetuximab for 72 h, determined by QPCR. (*p < 0.05 vs. vehicle control; **p < 0.01 vs. vehicle control; n.s., not significant; *n* = 3)

Next, we treated various ESCC cells with erlotinib or cetuximab. TE-1, TE-5, TE-11 and TE-11R cells exhibited high expression of E-cadherin and very low expression of vimentin, whereas TE-8 and HCE4 cells showed low expression of E-cadherin and high expression of vimentin (Fig. 3a). Therefore, we classified TE-1, TE-5, TE-11, and TE-11R cells as epithelial-like ESCC cells, and TE-8 and HCE4 cells as mesenchymal-like ESCC cells.

In agreement with the results of transformed-esophageal cells treated with EGFR inhibitors, the growth of epithelial-like ESCC cells (TE-1, TE-5, TE-11, and TE-11R) was significantly inhibited by treatment with erlotinib or cetuximab, but that of mesenchymal-like ESCC cells (TE-8 and HCE-4) was not affected (Fig. 3b). EdU assay showed that both erlotinib and cetuximab reduced the ratio of TE-5 (epithelial-like ESCC) cells in S phase but not HCE-4 (mesenchymal-like ESCC) cells (Additional file 3 Figure S3A). Moreover, the expression of involucrin mRNA and protein was increased by treatment with erlotinib (Fig. 3c and Additional file 3 Figure S3B) or cetuximab (Fig. 3d and Additional file 3 Figure S3C) in all epithelial-like ESCC cells but not mesenchymal-like ESCC cells (Figs. 3c, d, and Additional file 3 Figure S3B, C).

### Distinct effects of EGFR inhibitors on EGFR phosphorylation in epithelial- and mesenchymal-like esophageal cells

**Fig. 4:**
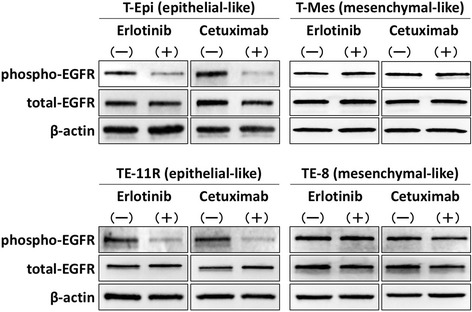
Distinct effects of EGFR inhibitors on EGFR phosphorylation in epithelial- and mesenchymal-like esophageal cells. Phosphorylated- and total-EGFR protein levels in epithelial-like esophageal cells (T-Epi and TE-11R) and mesenchymal-like esophageal cells (T-Mes and TE-8) treated with or without erlotinib or cetuximab for 24 h, determined by western blotting. Both erlotinib and cetuximab suppressed the phosphorylation of EGFR signaling in epithelial-like esophageal cells but not in mesenchymal-like esophageal cells

To explore the mechanisms underlying the differential effects of EGFR inhibitors on epithelial- and mesenchymal-like esophageal cells, we examined the inhibitory effects of EGFR signaling in epithelial-like (T-Epi and TE-11R) and mesenchymal-like (T-Mes and TE-8) esophageal cells treated with EGFR inhibitors. Notably, the phosphorylation of EGFR was suppressed by erlotinib or cetuximab in epithelial-like cells but was not fully suppressed in mesenchymal-like cells (Fig. 4).

### Invalidity of EGFR inhibitors on mesenchymal-like esophageal cells

**Fig. 5:**
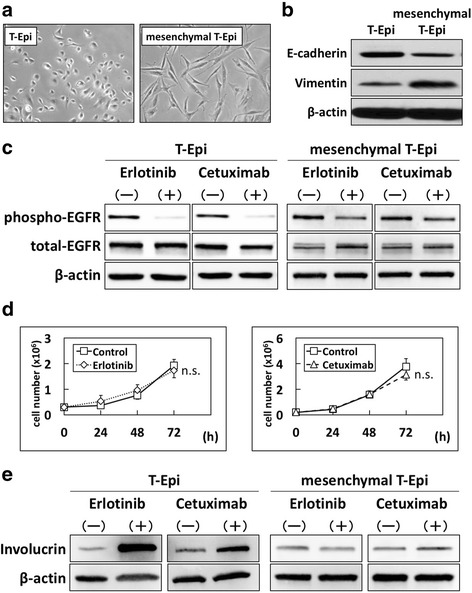
Resistance to EGFR inhibitors in mesenchymal T-Epi cells. T-Epi cells were treated with recombinant TGF-β1 (5 ng/mL) for 14 days to establish mesenchymal T-Epi cells. Mesenchymal T-Epi cells were cultured with normal KSFM medium (TGF-β1 free) for 24 h, and then parental T-Epi and mesenchymal T-Epi cells were treated with erlotinib or cetuximab. **a** Phase-contrast images of parental T-Epi cells and mesenchymal T-Epi cells. Note that T-Epi cells treated with TGF-β1 changed to spindle-shaped cells. **b** Protein levels of E-cadherin and vimentin in parental T-Epi and mesenchymal T-Epi cells, determined by western blotting. **c** Phosphorylated- and total-EGFR protein levels in parental T-Epi and mesenchymal T-Epi cells treated with erlotinib or cetuximab, determined by western blotting. The inhibitory effect of EGFR phosphorylation due to EGFR inhibitors was lower in mesenchymal T-Epi cells compared with parental T-Epi cells. **d** Cell growth of mesenchymal T-Epi cells treated with or without erlotinib or cetuximab. The results are presented as means ± SD (bars). (n.s., not significant, vs vehicle control; *n* = 3) Inhibition of cell growth was not observed in mesenchymal T-Epi cells. **e** Involucrin protein levels in parental T-Epi and mesenchymal T-Epi cells treated with or without erlotinib or cetuximab, determined by western blotting. The promotion of involucrin expression due to treatment with EGFR inhibitors was more suppressed in mesenchymal T-Epi cells compared with parental T-Epi cells

Thereafter, we examined whether epithelial- or mesenchymal-like phenotypes determine sensitivity to EGFR inhibitors. To test this, we treated T-Epi cells with recombinant TGF-β1 (5 ng/mL) for 14 days and established the cells as a mesenchymal phenotype (mesenchymal T-Epi cells; Fig. 5a), characterized by decreased E-cadherin expression and increased vimentin expression (Fig. 5b). Mesenchymal T-Epi cells were cultured with normal KSFM medium (TGF-β1 free) for 24 h, and parental T-Epi cells and mesenchymal T-Epi cells were treated with erlotinib or cetuximab. In agreement with the results using transformed-esophageal cells and ESCC cells, the inhibitory effects of EGFR inhibitors on EGFR phosphorylation were diminished in mesenchymal T-Epi cells, although EGFR phosphorylation was sufficiently suppressed in parental T-Epi cells (Fig. 5c). Furthermore, the inhibitory effects on cell growth (Fig. 5d) and the induction of involucrin expression (Fig. 5e) were not observed in mesenchymal T-Epi cells treated with erlotinib or cetuximab, which is in contrast to the responses of parental T-Epi cells.

### Antitumor effects of cetuximab on TE-11R- or TE-8-derived xenograft tumor

**Fig. 6:**
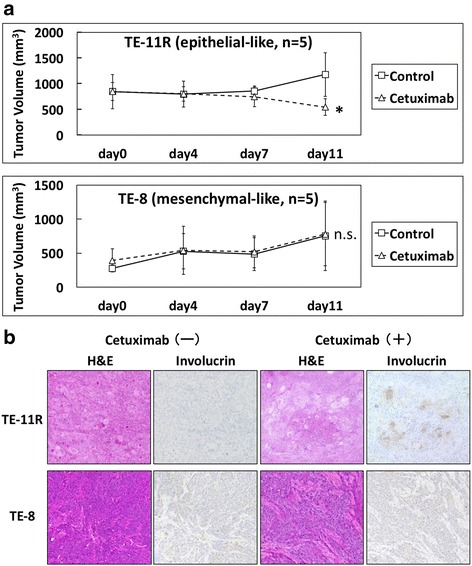
Distinct anti-tumor effects of cetuximab on xenograft tumors derived from epithelial- and mesenchymal-like ESCC cells. **a** Time-course volumes of TE-11R (epithelial-like ESCC cells) or TE-8 (mesenchymal-like ESCC cells)-derived xenograft tumors in vivo. TE-11R or TE-8-derived xenograft tumors were treated with intraperitoneal injection of cetuximab (50 μg/kg) or vehicle on days 0, 4, and 7. Cetuximab significantly inhibited tumor growth in TE-11R-derived xenograft tumors, but not in TE-8-derived xenograft tumors. (*p < 0.05 vs. vehicle control; n.s., not significant; *n* = 5). **b** Hematoxylin and eosin and immunohistochemical (involucrin) staining in serial sections. Keratin pearl formation, characterized by involucrin protein expression, was found in TE-11R-derived xenograft tumors treated with cetuximab but not the vehicle control. On the other hand, no expression was observed in TE-8-derived xenograft tumors in the presence or absence of cetuximab treatment

Xenograft tumors generated from TE-11R and TE-8 were treated with cetuximab to investigate the antitumor effects of EGFR inhibitors on ESCC. Treatment with cetuximab significantly suppressed cell growth in TE-11R (epithelial-like ESCC cells)-derived xenograft tumors (Fig. 6a). Additionally, keratin pearl formation in association with involucrin expression was observed in TE11R–derived xenograft tumors treated with cetuximab (Fig. 6b). In contrast, treatment with cetuximab did not suppress tumor growth (Fig. 6a) or affect involucrin expression (Fig. 6b) in TE-8 (mesenchymal-like ESCC cells)-derived xenograft tumors.

## Discussion

Here, we showed that treatment with EGFR inhibitors resulted in suppressed cell growth and increased squamous cell differentiation in epithelial-like esophageal cells but not mesenchymal-like esophageal cells. These phenomena were confirmed by immortalized- or transformed-esophageal cells and multiple ESCC cells. Notably, EGFR inhibitors suppressed the phosphorylation of EGFR in epithelial- but not mesenchymal-like esophageal cells. Moreover, mesenchymal T-Epi cells, which were generated from T-Epi cells by treating them with TGF-β1, showed resistance to EGFR inhibitors by circumventing the dephosphorylation of EGFR signaling. Our findings suggest that responses to EGFR inhibitors are different between epithelial- and mesenchymal-like esophageal cells.

In this study, we used two differently transformed-esophageal epithelial cells, T-Epi (epithelial-like phenotype) and T-Mes (mesenchymal-like phenotype) cells. T-Epi cells are transformed by EGFR and p53^R175H^ [19], and T-Mes cells by SV40 large T antigen and Ha-RasV12 [20]. Since these genes may influence the effects of EGFR inhibitors, we established cells with mesenchymal phenotypes (mesenchymal T-Epi cells) by treating T-Epi cells with TGF-β1 and compared the effects of EGFR inhibitors on parental T-Epi (epithelial-like) cells and mesenchymal T-Epi cells. Consequently, we were able to examine the effects of EGFR inhibitors on genetically identical cells with different phenotypes and reveal the different effects of EGFR inhibitors on epithelial- and mesenchymal-like esophageal cells.

In the present study, we showed that EGFR inhibitors promoted squamous cell differentiation in epithelial-like esophageal cells accompanied by increased expression of involucrin and CK13. The promotion of squamous cell differentiation was dramatically suppressed by γ-secretase inhibitors in epithelial-like cells (EPC2-hTERT, T-Epi, TE-1, and TE-5) (Additional file 4 Figure S4). These results are consistent with a previous report by Kolev et al. showing that EGFR inhibitors promoted cell differentiation in skin keratinocytes and cutaneous SCC cells via Notch signal activation [2]. We suspect that the cell-cell contact induced by EGFR inhibitors might be involved in the promotion of squamous cell differentiation because it increases cell-cell interactions such as Notch signaling [29], which may help to promote squamous cell differentiation.

In this study, EGFR inhibitors showed antitumor effects in epithelial-like ESCC cells but not in mesenchymal-like ESCC cells. These effects were also reported in other malignancies such as head and neck squamous cell carcinoma, and non-small cell lung carcinoma [30–32]; however, no articles describe the underlying mechanism or analyze the phenomenon from the perspective of EGFR dephosphorylation. As EGFR signal activation is reported to be closely associated with chemoresistance or poor prognosis in squamous cell carcinomas as well as other malignancies [33–35], effective suppression of EGFR signaling is considered to be important for the treatment of these cancers.

We found here for the first time that treatment with EGFR inhibitors dephosphorylated EGFR signaling in epithelial-like esophageal cells but not in mesenchymal-like esophageal cells. Similarly, treatment with rEGF enhanced the EGFR phosphorylation in epithelial-like esophageal cells but not in mesenchymal-like esophageal cells. As EGFR was originally identified as a growth factor receptor in the “epidermis” [36, 37], we speculate that EGFR phosphorylation plays a role only in epithelial-like esophageal cells, and not in mesenchymal-like esophageal cells. Consistently, esophageal fibroblast cells, which are the mesenchymal cells, were not affected by treatment with EGFR inhibitors or rEGF (Additional file 5 Figure S5). Taken together, the failure of EGFR dephosphorylation by EGFR inhibitors in mesenchymal-like esophageal cells is thought to be an important mechanism of resistance to EGFR inhibitors.

Given the results of this study, EGFR inhibitors are considered effective for epithelial-like ESCC cells but ineffective for mesenchymal-like ESCC cells. However, it is unclear whether ESCC patients can be divided into two groups based on the cells being epithelial- or mesenchymal-like. Recently, a study on the pathology of ESCC patients revealed that several phenotypes such as epithelial-like (E-cadherin: positive/vimentin- or N-cadherin: negative), mesenchymal-like (E-cadherin: negative/vimentin- or N-cadherin: positive), hybrid type (E-cadherin: positive/vimentin- or N-cadherin: positive), and null type (E-cadherin: negative/vimentin- or N-cadherin: negative) exist in ESCC patients [38]. Given that there have been no clinical investigations into the effects of EGFR inhibitors on ESCC patients based on epithelial- or mesenchymal-like phenotype, further clinical research is required.

One limitation of our study was that we were unable to elucidate the mechanism by which EGFR inhibitors fail to induce cell adhesion and EGFR dephosphorylation in mesenchymal-like esophageal cells. Further study is needed to elucidate this mechanism. In addition, we did not discover an alternative therapeutic strategy for mesenchymal-like esophageal cells. Furthermore, the synergistic effects of radiotherapy or other anticancer drugs on squamous cell differentiation due to EGFR inhibitors should be examined in the future.

## Conclusions

**Fig. 7.**
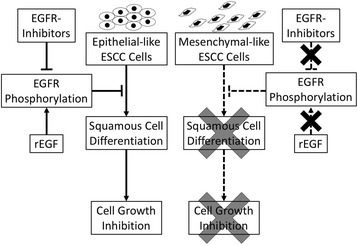
Schematic summary of our study. We showed the distinct effects of EGFR inhibitors on epithelial-like and mesenchymal-like ESCC cells. In epithelial-like ESCC cells, suppression of EGFR signaling due to EGFR inhibitors resulted in the promotion of squamous cell differentiation and cell growth inhibition; conversely, activation of EGFR phosphorylation due to rEGF suppressed squamous cell differentiation. In contrast, neither EGFR inhibitors nor rEGF affected EGFR phosphorylation in mesenchymal-like ESCC cells, and thereby mesenchymal-like ESCC cells are considered to be resistant to EGFR inhibitors.

We revealed that the factors determining the therapeutic effects of EGFR inhibitors in ESCC cells are the phenotypes representing the epithelial-like or mesenchymal-like cells. In epithelial-like ESCC cells, EGFR inhibition promotes squamous cell differentiation through suppression of EGFR phosphorylation, and conversely, activation of EGFR phosphorylation suppresses squamous cell differentiation. Importantly, promotion of squamous cell differentiation results in tumor cell growth inhibition. In contrast, EGFR signaling is affected by neither EGFR inhibitors nor rEGF in mesenchymal-like ESCC cells, and thereby squamous cell differentiation and tumor cell growth inhibition do not occur in mesenchymal-like ESCC cells treated with EGFR inhibitors (Fig. 7).

Thus, the differential effects of EGFR inhibitors on EGFR phosphorylation are considered to be the underlying mechanisms that determine the response to EGFR inhibitors. Our findings provide novel, mechanistic insights into the effects of EGFR inhibitors on ESCC.

## Additional files

Additional file 1: Figure S1. Effects of EGFR inhibition or activation on immortalized-human esophageal epithelial cells. EPC2-hTERT, an immortalized-human esophageal epithelial cell line, was treated with erlotinib (1 μM) or cetuximab (100 μg/mL). (A) Cytokeratin13 (CK13) mRNA expression levels in EPC2-hTERT cells treated with or without erlotinib or cetuximab for 72 h determined by QPCR. The gene for β-actin served as an internal control. (**p < 0.01 vs. vehicle control; *n* = 3). (B) CK13 mRNA expression levels in EPC2-hTERT cells treated with recombinant EGF (rEGF) for 48 h determined by QPCR. The gene for β-actin served as an internal control. (**p < 0.01 vs. vehicle control; *n* = 3). (JPEG 130 kb)

Additional file 2: Figure S2. Differential effects of EGFR inhibitors in epithelial- and mesenchymal-like transformed-human esophageal epithelial cells. (A) CK13 mRNA expression levels in T-Epi or T-Mes cells treated with or without erlotinib or cetuximab determined by QPCR. (**p < 0.01 vs. vehicle control; n.s. represents not significant; *n* = 3). (B) Phosphorylated- and total-EGFR protein level in T-Epi and T-Mes cells treated with human recombinant EGF (rEGF) and erlotinib for 24 h determined by western blotting. (C) Involucrin mRNA expression levels in T-Epi and T-Mes cells treated with rEGF and erlotinib for 24 h determined by QPCR. (**p < 0.01 rEGF(+)/erlotinib(−) vs. vehicle control, rEGF(+)/erlotinib(+) vs. rEGF(+)/erlotinib(−); n.s. represents not significant; *n* = 3). (D) Involucrin protein production levels in T-Epi and T-Mes cells treated with rEGF and erlotinib for 24 h determined by western blotting. (E) CK13 mRNA expression levels in T-Epi and T-Mes cells treated with rEGF and erlotinib for 24 h determined by QPCR. (**p < 0.01 rEGF(+)/erlotinib(−) vs. vehicle control, rEGF(+)/erlotinib(+) vs. rEGF(+)/erlotinib(−); n.s. represents not significant; *n* = 3) (JPEG 542 kb)

Additional file 3: Figure S3. Differential effects of EGFR inhibitors in epithelial- and mesenchymal-like ESCC cells. (A) Cell cycle of epithelial-like TE-5 cells and mesenchymal-like HCE-4 cells treated with or without erlotinib or cetuximab for 72 h, analyzed by EdU assay. Cells in S phase are plotted in p3, and cells in other phases in p2. The experiments were conducted in triplicate, and results are represented as means ± SD. Representative data are shown. (*n* = 3). (B) Involucrin protein levels in ESCC cells treated with erlotinib for 72 h determined by western blotting. Densitometry values are noted (C) Involucrin protein levels in ESCC cells treated with cetuximab for 72 h determined by western blotting. Densitometry values are noted. (JPEG 579 kb)

Additional file 4: Figure S4. Effects of γ-secretase inhibitors on squamous cell differentiation in epithelial-like esophageal cells treated with EGFR inhibitors. Involucrin mRNA expression levels in (A) EPC2-hTERT cells, (B) T-Epi cells, and (C) TE-1 and TE-5 cells determined by QPCR. Cells were treated with DAPT (10 μM) and/or EGFR inhibitors (erlotinib [1 μM] or cetuximab [100 μg/mL]) for 72 h. (**p < 0.01 vs. vehicle control; n.s., not significant; *n* = 3). (JPEG 456 kb)

Additional file 5: Figure S5. Effects of EGFR inhibitors on esophageal fibroblast cells. (A) E-cadherin and vimentin protein production levels in FEF3 cells (human fetal esophageal mesenchymal cells) determined by western blotting. EPC2-hTERT cells were used as a control. (B) Cell growth of FEF3 cells treated with or without erlotinib or cetuximab. Results are represented as means ± SD (bars). (n.s., not significant, vs vehicle control; *n* = 3) No inhibition of cell growth was observed in FEF3 cells treated with erlotinib or cetuximab. (C) Phosphorylated- and total-EGFR protein level in FEF3 cells treated with human recombinant EGF (rEGF) for 24 h determined by western blotting. Untreated EPC2-hTERT cells were used as a positive control. rEGF did not activate EGFR signaling in FEF3 cells. (D) Involucrin protein production levels in FEF3 cells treated with or without erlotinib or cetuximab for 72 h determined by western blotting. Untreated EPC2-hTERT cells were used as positive controls. Treatment with EGFR inhibitors did not increase involucrin protein production levels in FEF3 cells. (E) Phosphorylated- and total-EGFR protein levels in FEF3 cells treated with or without erlotinib or cetuximab for 72 h determined by western blotting. Untreated EPC2-hTERT cells were used as a positive control. Neither erlotinib nor cetuximab suppressed the phosphorylation of EGFR signaling in FEF3 cells (mesenchymal-like cells). (JPEG 381 kb)

## Abbreviations

CK13: Cytokeratin13
EGFR: Epidermal growth factor receptor
ESCC: Esophageal squamous cell carcinoma
H&E: Hematoxylin and eosin
KSFM: Keratinocyte serum-free medium
rEGF: Recombinant EGF
RT-PCR: Real-time reverse transcriptase polymerase chain reaction
