# Supplementary figures and images for: Distinct effects of EGFR inhibitors on epithelial- and mesenchymal-like esophageal squamous cell carcinoma cells
Source: J Exp Clin Cancer Res. 2017 Aug 1;36:101. doi: 10.1186/s13046-017-0572-7 (PMC5540425; doi:10.1186/s13046-017-0572-7)

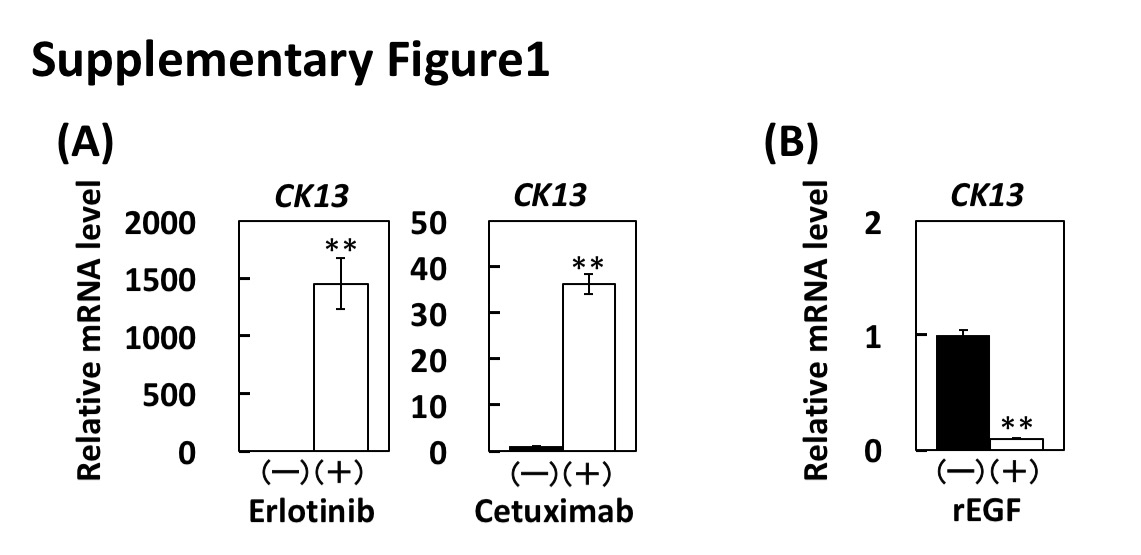

Supplement: Supplementary file 1 — Effects of EGFR inhibition or activation on immortalized-human esophageal epithelial cells. EPC2-hTERT, an immortalized-human esophageal epithelial cell line, was treated with erlotinib (1 μM) or cetuximab (100 μg/mL). (A) Cytokeratin13 (CK13) mRNA expression levels in EPC2-hTERT cells treated with or without erlotinib or cetuximab for 72 h determined by QPCR. The gene for β-actin served as an internal control. (**p < 0.01 vs. vehicle control; n = 3). (B) CK13 mRNA expression levels in EPC2-hTERT cells treated with recombinant EGF (rEGF) for 48 h determined by QPCR. The gene for β-actin served as an internal control. (**p < 0.01 vs. vehicle control; n = 3). (JPEG 130 kb) [file 13046_2017_572_MOESM1_ESM.jpg]

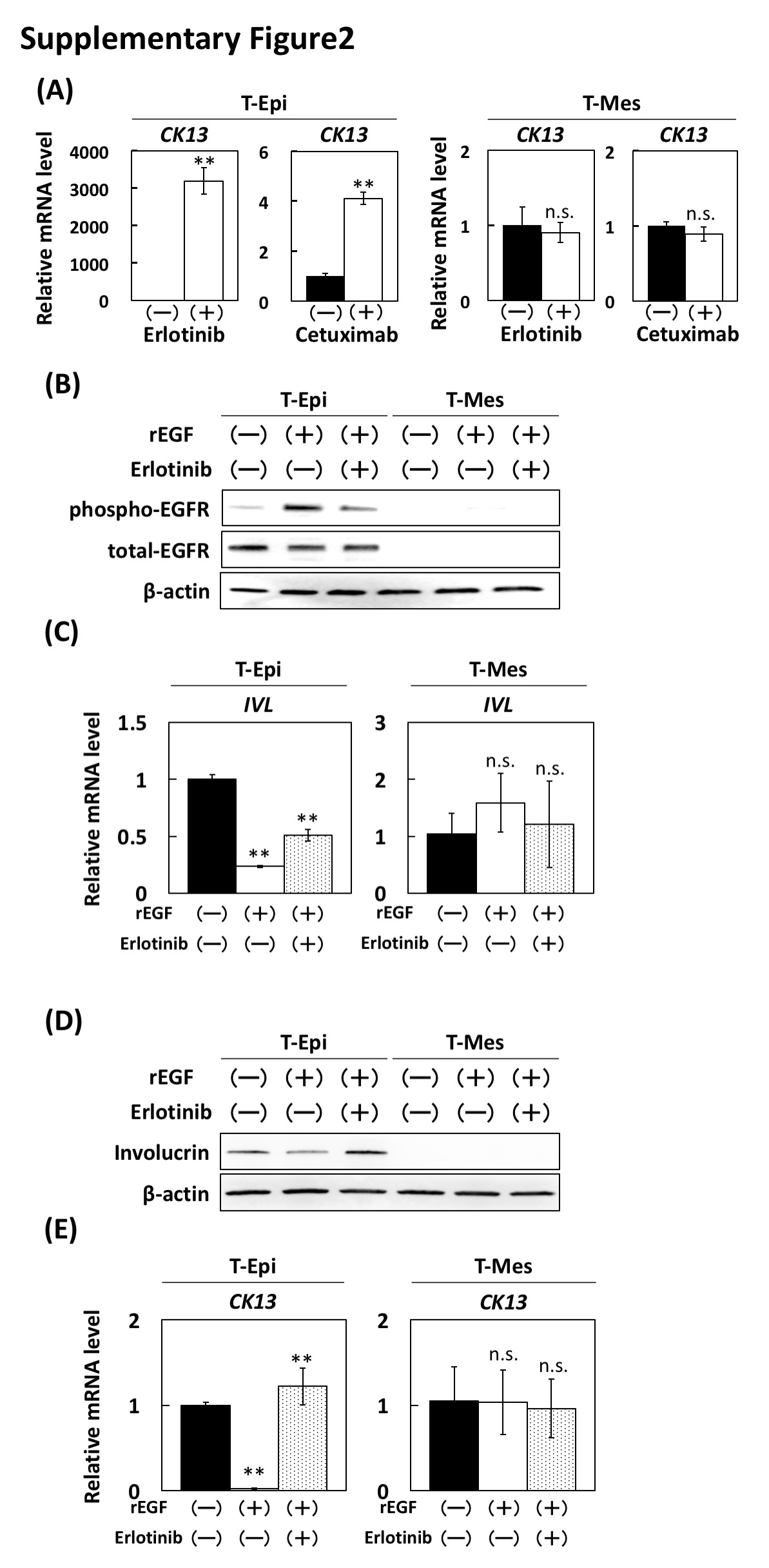

Supplement: Supplementary file 2 — Differential effects of EGFR inhibitors in epithelial- and mesenchymal-like transformed-human esophageal epithelial cells. (A) CK13 mRNA expression levels in T-Epi or T-Mes cells treated with or without erlotinib or cetuximab determined by QPCR. (**p < 0.01 vs. vehicle control; n.s. represents not significant; n = 3). (B) Phosphorylated- and total-EGFR protein level in T-Epi and T-Mes cells treated with human recombinant EGF (rEGF) and erlotinib for 24 h determined by western blotting. (C) Involucrin mRNA expression levels in T-Epi and T-Mes cells treated with rEGF and erlotinib for 24 h determined by QPCR. (**p < 0.01 rEGF(+)/erlotinib(−) vs. vehicle control, rEGF(+)/erlotinib(+) vs. rEGF(+)/erlotinib(−); n.s. represents not significant; n = 3). (D) Involucrin protein production levels in T-Epi and T-Mes cells treated with rEGF and erlotinib for 24 h determined by western blotting. (E) CK13 mRNA expression levels in T-Epi and T-Mes cells treated with rEGF and erlotinib for 24 h determined by QPCR. (**p < 0.01 rEGF(+)/erlotinib(−) vs. vehicle control, rEGF(+)/erlotinib(+) vs. rEGF(+)/erlotinib(−); n.s. represents not significant; n = 3) (JPEG 542 kb) [file 13046_2017_572_MOESM2_ESM.jpg]

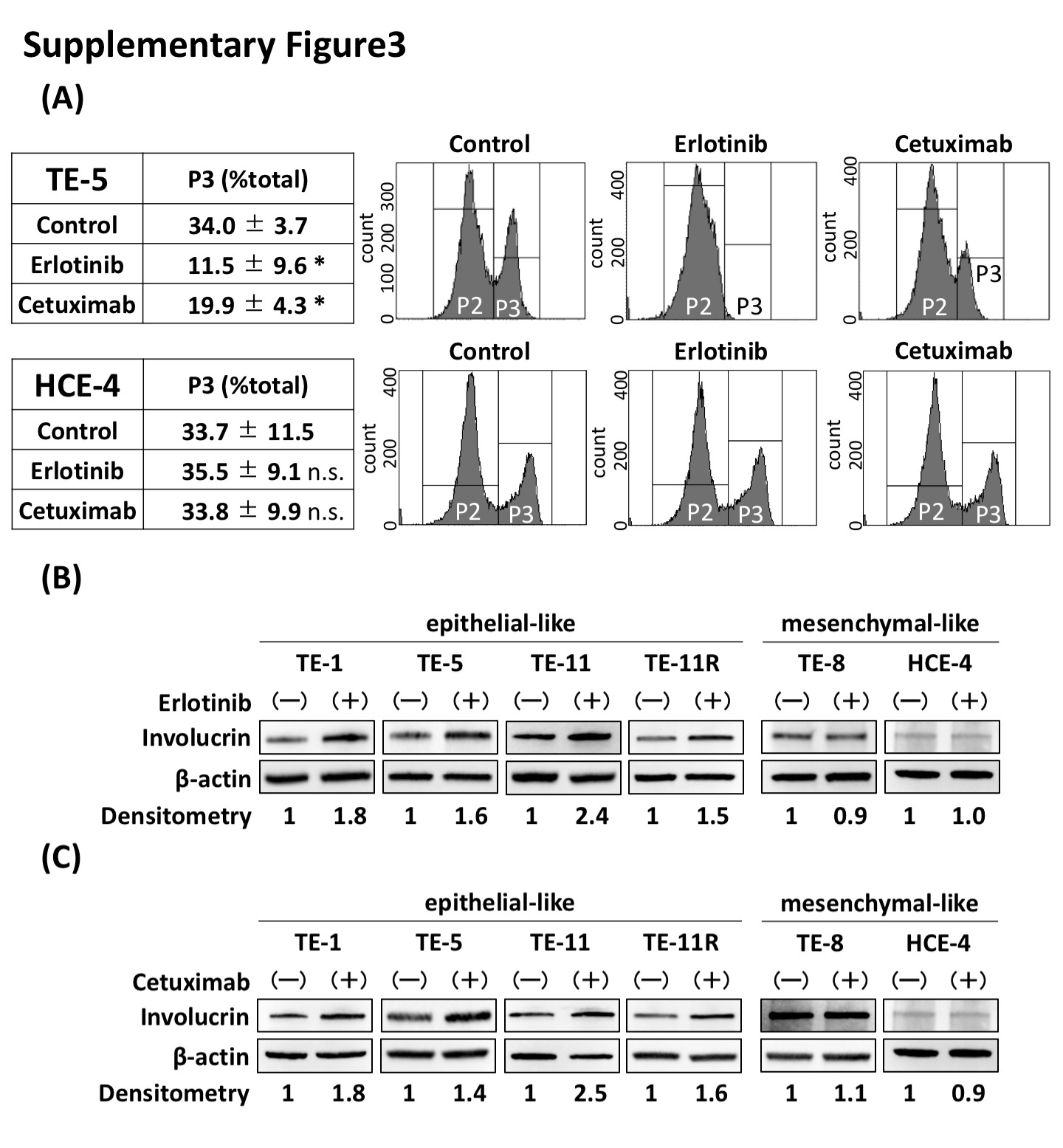

Supplement: Supplementary file 3 — Differential effects of EGFR inhibitors in epithelial- and mesenchymal-like ESCC cells. (A) Cell cycle of epithelial-like TE-5 cells and mesenchymal-like HCE-4 cells treated with or without erlotinib or cetuximab for 72 h, analyzed by EdU assay. Cells in S phase are plotted in p3, and cells in other phases in p2. The experiments were conducted in triplicate, and results are represented as means ± SD. Representative data are shown. (n = 3). (B) Involucrin protein levels in ESCC cells treated with erlotinib for 72 h determined by western blotting. Densitometry values are noted (C) Involucrin protein levels in ESCC cells treated with cetuximab for 72 h determined by western blotting. Densitometry values are noted. (JPEG 579 kb) [file 13046_2017_572_MOESM3_ESM.jpg]

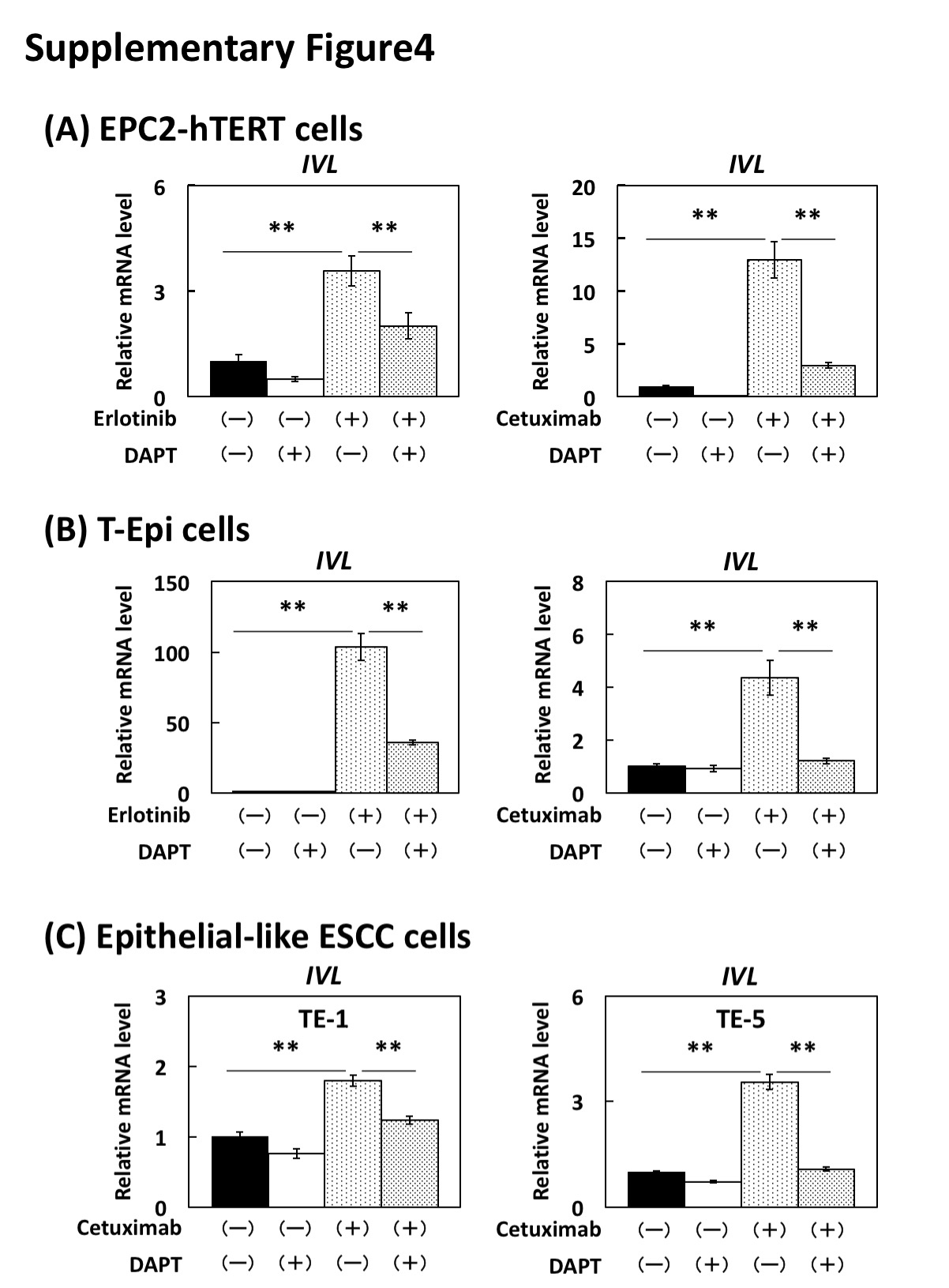

Supplement: Supplementary file 4 — Effects of γ-secretase inhibitors on squamous cell differentiation in epithelial-like esophageal cells treated with EGFR inhibitors. Involucrin mRNA expression levels in (A) EPC2-hTERT cells, (B) T-Epi cells, and (C) TE-1 and TE-5 cells determined by QPCR. Cells were treated with DAPT (10 μM) and/or EGFR inhibitors (erlotinib [1 μM] or cetuximab [100 μg/mL]) for 72 h. (**p < 0.01 vs. vehicle control; n.s., not significant; n = 3). (JPEG 456 kb) [file 13046_2017_572_MOESM4_ESM.jpg]

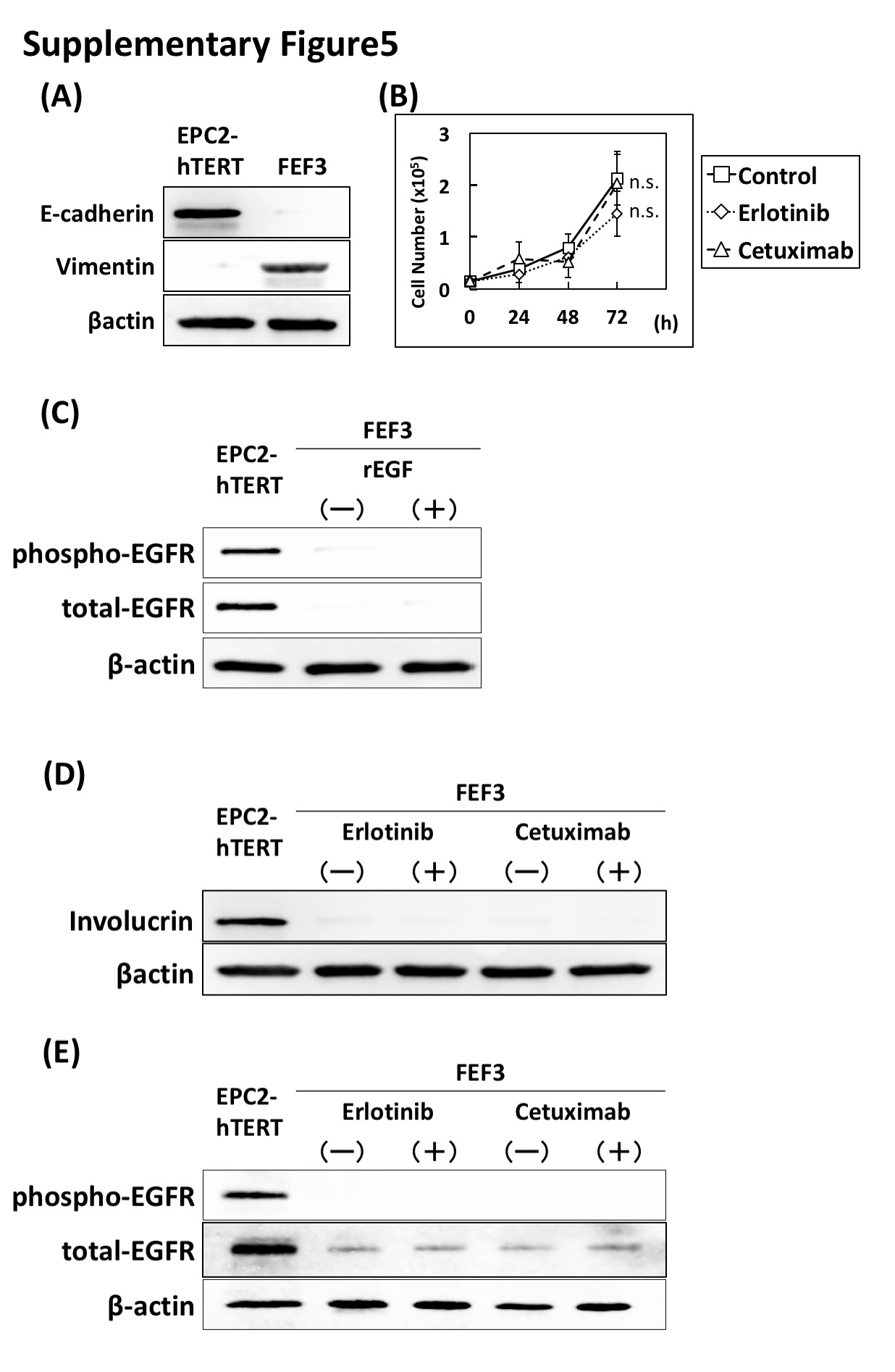

Supplement: Supplementary file 5 — Effects of EGFR inhibitors on esophageal fibroblast cells. (A) E-cadherin and vimentin protein production levels in FEF3 cells (human fetal esophageal mesenchymal cells) determined by western blotting. EPC2-hTERT cells were used as a control. (B) Cell growth of FEF3 cells treated with or without erlotinib or cetuximab. Results are represented as means ± SD (bars). (n.s., not significant, vs vehicle control; n = 3) No inhibition of cell growth was observed in FEF3 cells treated with erlotinib or cetuximab. (C) Phosphorylated- and total-EGFR protein level in FEF3 cells treated with human recombinant EGF (rEGF) for 24 h determined by western blotting. Untreated EPC2-hTERT cells were used as a positive control. rEGF did not activate EGFR signaling in FEF3 cells. (D) Involucrin protein production levels in FEF3 cells treated with or without erlotinib or cetuximab for 72 h determined by western blotting. Untreated EPC2-hTERT cells were used as positive controls. Treatment with EGFR inhibitors did not increase involucrin protein production levels in FEF3 cells. (E) Phosphorylated- and total-EGFR protein levels in FEF3 cells treated with or without erlotinib or cetuximab for 72 h determined by western blotting. Untreated EPC2-hTERT cells were used as a positive control. Neither erlotinib nor cetuximab suppressed the phosphorylation of EGFR signaling in FEF3 cells (mesenchymal-like cells). (JPEG 381 kb) [file 13046_2017_572_MOESM5_ESM.jpg]
